# Jerked Beef: Chemical Composition and Desalting Techniques

**DOI:** 10.3390/foods14213745

**Published:** 2025-10-31

**Authors:** Maria do Desterro Pereira Ferreira Ibiapina, Maria Eduarda Corino de Melo, Márcio Antônio Mendonça, Frederico Lopes da Silva, Myller de Sousa Tonhá, Raquel Braz Assunção Botelho

**Affiliations:** 1 Human Nutrition Graduate Program, University of Brasília, Brasília 70910-900, Brazil; mdesterrop@gmail.com; 2 Undergraduate Program, Nutrition Department, University of Brasília, Brasília 70910-900, Brazil; meduardacorino@gmail.com; 3 College of Agronomy and Veterinary Medicine, University of Brasília, Brasília 70910-900, Brazil; marcioamen@gmail.com; 4 Federal Institute of Brasília, Planaltina-DF Campus, Planaltina 73380-900, Brazil; frederico.lopes@ifb.edu.br; 5 Graduate Program in Geology, Institute of Geosciences, University of Brasília, Brasília 70910-900, Brazil; myllerquimico@gmail.com; 6 Nutrition Departament, University of Brasília, Brasília 70910-900, Brazil

**Keywords:** desalting, sodium, oxidative stability, meat processing, reduction

## Abstract

The present study aims to compare the chemical composition of samples of jerked beef commercialized in Brasilia, Brazil, subjected to diverse desalting techniques (room temperature, refrigerated, and heat desalting). This experimental study was divided into five steps: determination of desalting techniques, chemical composition, determination of titratable acidity and pH, sodium analysis of the samples, and statistical analysis. The control samples showed high sodium levels (>6000 mg/100 g), confirming the need for desalting to ensure suitability for consumption. Desalting at room temperature was the most efficient, reducing sodium content by up to 76%, followed by refrigeration (67–74%) and the heat method (52–58%). It was also observed that the desalting technique significantly affects the chemical composition. Desalting at room temperature and under refrigeration increased moisture (54.12→73.82 g/100 g) and reduced proteins (23.50→18.70 g/100 g) and lipids (3.70→3.00 g/100 g) through a dilution effect, while desalting in heat concentrated solids, increasing protein (31.29 g/100 g), lipids (4.19 g/100 g), and lipid oxidation (TBARS = 91.79 µmol MDA/kg) in comparison to control samples (38.63 µmol MDA/kg). Acidity and pH showed minor variations but correlated with lipid oxidation processes. Although no technique eliminates excess sodium, the results reinforce that desalting at room temperature offers the best balance between sodium reduction and preservation of the product’s nutritional quality and oxidative stability, making it the most suitable method for use in restaurants and at home.

## 1. Introduction

Meat is considered a noble food, rich in high-quality proteins, vitamins, and minerals, which meet many of human nutritional needs [1]. Beef is considered essential in the preparation of meals in different global cuisines, including popular Brazilian cuisine, which explains the interest in studies on this product [2,3,4].

Since it contains around 70% water, a fundamental component of physical-chemical, biochemical, and microbiological changes that cause food deterioration, preservation methods such as dehydration are used on meat to increase its shelf life [5,6].

Salting is a traditional preservation process, and in many cases, the resulting product undergoes fermentation and is then consumed as a new product [7]. Some authors define salting as a curing process that imparts specific sensory properties to the product. Preservation is achieved through reactions between sodium chloride (NaCl) and free water molecules present in the food, via osmotic dehydration, which reduces microbial activity and increases chemical and enzymatic reactions [8,9]. In addition to this effect, adding salt enhances sensory attributes, generating a pleasant aroma and flavor and thereby increasing attractiveness [10,11]. Processed meats have an extended shelf life and are affordable and available in various markets throughout the year. Miller et al. [12] reported an average daily consumption of processed meat of 37 g in the Latin America and Caribbean region.

According to Neto et al. [13], in their systematic review, salting and dissection are the main processing techniques for meats. In Brazil, there are three different types of processed meat: charqui, jerked beef, and sun-dried meat. Charqui is produced by curing meat with salt and then exposing it to the sun. For jerked beef, besides salt, the meat is cured, and the sun-dried technique is a more artisanal approach that uses less salt and exposes the meat to the sun. The most common product in the Brazilian market is jerked beef, primarily because it is sold in vacuum packages, making it easier to sell in supermarkets.

Despite being a key ingredient in extending shelf life, the introduction of salt into the human diet led to the replacement of a primitive diet rich in potassium and low in sodium with one that is low in potassium and high in sodium [14]. Processes such as salting, as well as the indiscriminate use of sodium in preparations, lead to excessive intake, being the main cause of the development of chronic diseases [15]. Excess sodium in the diet is one of the main risk factors for hypertension and is directly associated with cardiovascular and kidney events [16,17,18].

Recent epidemiological studies indicate that average sodium consumption in Brazil remains above the World Health Organization (WHO) recommendations, representing a relevant factor for cardiovascular disease. The average sodium intake among Brazilian adults is 2432 mg/day (95% CI: 1902–3074 mg), and approximately 61% of the population exceeds the tolerable upper limit [19]. Similar results were reported by Nilson et al. [20], who observed an increase in the average sodium availability in Brazilian households, from 3.9 g to 4.7 g/2000 kcal, between 2002 and 2003 and 2017–2018, demonstrating an upward trend in the consumption of industrialized foods as the primary dietary source of sodium. Thus, in a complementary manner, Mill et al. [15] estimated the average salt intake at 9.34 g/day (≈3700 mg sodium), reinforcing the persistence of high levels in the Brazilian diet. Therefore, these studies show that, despite advances in public policies for food reformulation and nutritional labeling, Brazil still has sodium intake above the WHO limit (2000 mg/day). With more restrictive consumption targets, strategies to reduce sodium intake are crucial for mitigating the risk of disease. Therefore, salty products need to be desalted before consumption.

Jerked beef, a salt-cured meat product available on the Brazilian market, requires desalting before consumption. This process, in addition to modifying its nutritional value, can also affect its fatty acid content [21,22,23]. Despite its widespread use in meal production in Brazil, there is little interest in the literature on jerked beef, its standardization, and the impact of different desalting techniques. No prior study has compared desalting efficiency under room, refrigeration, and heat conditions in jerked beef. Therefore, the present study aims to compare the chemical composition of jerked beef subjected to diverse desalting techniques.

## 2. Materials and Methods

This experimental study was divided into five steps: (1) determination of desalting techniques of jerked beef; (2) chemical composition of the samples; (3) determination of titratable acidity and pH; (4) sodium analysis of the samples; (5) statistical analysis. Desalted jerked beef samples, stored at room temperature, refrigerated, and subjected to heat treatments, were stored in freezers at −18 °C and analyzed within 90 days. Salted samples were used as the control treatment and stored at −18 °C in freezers.

### 2.1. Determination of Desalting Techniques of Jerked Beef

Jerked beef from three different producers was purchased at supermarkets in Brasilia, Federal District. For each producer, researchers bought three different batches. Each package of jerked beef weighed 500 g, and for all analyses, 12 packages were purchased.

For the pre-preparation technique, cuts of similar size to those used in restaurants were used: cubes with edges of approximately 2 cm each, weighing approximately 25 g. When preparing the cuts, parts that presented only fat and no meat were discarded.

Since the focus was on analyzing meats as they are prepared in restaurants in Brazil, three desalting techniques were selected because they are the most common methods for reducing sodium chloride content.

Desalting was performed according to the criteria established in CVS, Brazil [24], and the following treatments were applied: DRT—desalting at room temperature (23 °C), DFT—desalting at refrigerator temperature (5 °C), and DHT—desalting at heat temperature. The water ratio for all three methods was 1:3 (meat-to-water).

For desalting at room temperature and in the fridge, 50 g of each sample was placed in a container with water at room temperature (23 °C) and refrigerated (5 °C), respectively. After the first 7 h of desalting, the water was changed, and the entire process was repeated. To determine the time before changing the water, a previous test was conducted with jerked beef samples. During the determination of desalination time using water exchange, the electrical conductivity of the water was measured every hour for 12 h using a multiparameter device. Simultaneously, 0.05 mL aliquots were collected, diluted (1:100), and analyzed in an ICP-QES spectrometer (5100, Agilent Technologies, Santa Clara, CA, USA) for sodium quantification. After 7 h, the water in the samples was changed, and the procedure was repeated, allowing the calculation in mg/100 g, according to the criteria of CVS, Brazil [24].

In heat desalting, water was poured into a pan, and once it boiled, the heat was turned off. The meat was then immersed in the boiling water. Then, using a glass thermometer, the water temperature was measured, and when it reached 50 °C, the meat was removed from the water to prevent the proliferation of mesophilic bacteria [25,26]. Subsequently, the water was changed, and the entire process was repeated. Studies show that selecting a temperature of 50 °C reduces microbial levels without compromising the food’s physicochemical characteristics, and it is considered a safe parameter for immersion or thermal desalting processes [27,28].

Researchers decided to change water only once for the three desalting techniques because restaurants do not have time to prepare their ingredients to produce meals, and also because of sustainability (less water waste).

### 2.2. Analysis of Sodium Content in Jerked Beef Samples

The sodium content was determined using an ICP-OES spectrometer (5100, Agilent Technologies, Santa Clara, CA, USA) according to the AOAC method [29]. A six-point calibration curve (p0 = 0; p6 = 6.32209) was generated using the Sodium Standard for ICP 10 g/L Na in nitric acid (Sigma-Aldrich, St. Louis, MO, USA).

For the meat analysis, 1 g aliquots of the control meat samples (from different batches and producers) and 1 g of the desalted meat from each treatment were collected and placed in porcelain crucibles for incineration in a muffle furnace (EDG, 1800, Sao Paulo, Brazil) at 550 °C. After cooling, the contents were subjected to acid digestion with 50 mL of 10% HCl, stirred with a magnetic stirrer until complete dissolution was achieved. The solution was transferred to a 100 mL volumetric flask, and the volume was brought to 100 mL with distilled water. From this solution, a 0.05 mL aliquot was collected and placed in a Falcon tube for dilution with 5 mL of distilled water, resulting in a 1:100 dilution ratio. The solubilized meat samples were evaluated directly in the Falcon tubes, and the sodium concentration, expressed in milligrams per 100 g, was calculated based on the reading results.

### 2.3. Jerked Beef Chemical Composition

Control samples from three different producers, each from three different batches, were analyzed in triplicate for moisture [29], ashes [30], proteins [31], lipids (extracted using a Soxhlet apparatus with an Ankom Model XT10 extractor [32], and lipid oxidation. Researchers calculated carbohydrates by difference, considering 100—(weight in grams (fat + protein + ash)) in 100 g of jerked beef.

#### 2.3.1. Moisture Determination

Moisture content was determined according to method [29] by direct oven drying (Marconi—MA035/1) at 105 °C. On a scale (Shimadzu—ATX224R, Kyoto, Japan), 5 g of the sample was weighed in a previously weighed porcelain capsule. The sample was then placed in an oven at 105 °C until it reached a constant weight. After this time, the capsule and sample assembly were cooled in a desiccator and subsequently weighed for calculation.

#### 2.3.2. Ashes

Ash content was determined using the incineration residue method [30], expressed as a percentage (m/m). The porcelain capsule, previously heated in a muffle furnace (Linn Elektro Therm, Eschenfelden, Germany) to 550 °C and then cooled to room temperature, was weighed. One gram of the sample was weighed into this capsule. The sample was then placed in the muffle furnace and heated to 550 °C for 5 h. The loss of mass will correspond to the organic matter content of the product, as it will be carbonized entirely at this temperature. After this time, the capsule and samples were cooled in a desiccator (Sao Paulo, Brazil), and the entire set was weighed for calculation.

#### 2.3.3. Protein

Protein determination was performed using the Kjeldahl method, as described by AACC [31]. Approximately 0.3 g of the sample was digested with concentrated sulfuric acid (Synth) and a catalytic mixture, followed by distillation in the presence of 40% NaOH (Neon, São Paulo, Brazil) and ammonia capture in a 4% boric acid (Vetec, Rio Janeiro, Brazil) solution. Quantification was performed by titration with 0.1 N HCl (Synth), using methyl red and methylene blue (Sigma-Aldrich) as indicators.

#### 2.3.4. Lipids

To determine the lipid content of the samples, the petroleum extraction method [32], adapted for the Ankom XT10, was used, using the ANKOM^®^ extractor (ANKROM Technology, Macedon, NY, USA). Each sample, weighing 1 g, was placed in XT10 extraction filters and sealed by direct heat. The samples were then oven-dried (Marconi—MA035/1, Sao Paulo, Brazil) at 105 °C for 3 h and subsequently cooled in a glass desiccator (Sao Paulo, Brazil) with silica gel. After cooling, the samples were placed in the equipment, where they remained for 50 min in contact with petroleum ether, which dissolved and extracted the lipids.

#### 2.3.5. Carbohydrates

The carbohydrate content was determined by subtracting the sum of the sample’s protein, lipid, moisture, and ash contents from its total weight.

#### 2.3.6. Lipid Oxidation

Five grams of each sample were mixed with 15 mL of 7.5% TCA (trichloroacetic acid, Dinamica) and homogenized for 1 min. The mixture was then filtered using a 100 mL beaker with qualitative paper. From the filtered, 5 mL was transferred to a Falcon tube (15 mL) and 5 mL of TBA (thiobarbituric acid, Sigma-Aldrich) was added. After vigorous agitation, tubes were placed in a bain-marie for 40 min at 100 °C. Samples were cooled for 15 min, and absorbance was measured at 532 and 600 nm (Spectrophotometer Shimadzu, Kyoto, Japan). The methodology used for lipid oxidation analysis was the thiobarbituric acid reactive substances (TBARS) method, as described by Malsen, Sorensen, Skibsted, and Bertelsen [33].

### 2.4. Determination of Titratable Acidity and pH

As described by AOAC 981,12 [34], pH was determined by weighing 5 g of each sample and adding 50 mL of water. Using a glass rod, the solution was homogenized through rotational movements. After this, the electrode of a benchtop pH meter (Digimed—DM21, Sao Paulo, Brazil) was inserted into the mixture to measure the pH.

Acidity was performed as described by AOAC 942.15 [34]. On a scale (Shimadzu—ATX224R, Kyoto, Japan), 5 g of the sample was weighed into a previously weighed beaker, and then 50 mL of water was added. Using a magnetic bar and magnetic stirrer, the solution was homogenized, and the electrode of a benchtop pH meter (Digimed—DM21, Sao Paulo, Brazil) was inserted. The solution was titrated with 0.1 M sodium hydroxide solution (Synth, Sao Paulo, Brazil).

### 2.5. Statistical Analysis

One-way analyses of variance (ANOVA) with Tukey post hoc tests were performed in Microsoft Excel (Office 365—version 16) to assess chemical composition, sodium content, pH, and titratable acidity. Using a 95% confidence level, the data were analyzed and considered statistically significant when *p* < 0.05.

## 3. Results

Three different producers of jerked beef were selected for this study: Brands A, B, and C. For each Brand, three different batches were purchased to better reflect each production method of each producer more accurately.

### 3.1. Analysis of Sodium Content in Jerked Beef Samples

Table 1 presents the sodium content of control samples and after desalting for the three brands. The results show the mean and standard deviation of the batches for each Brand. Also, the percentage of sodium reduction when compared to the control is presented for each desalting technique.

Although Brand B had the highest sodium content (6538.6 mg/100 g), all brands did not differ statistically (*p* > 0.005). The Brand with the lowest sodium content across all the desalting techniques was Band A, with room temperature being the lowest (1527.4 mg/100 g). For all brands, room-temperature desalting resulted in the greatest sodium reduction compared to control samples. For Brand A, there was no difference between room temperature and refrigerated conditions (*p* > 0.005), but this was not observed for Brands B and C. Heat desalting resulted in the lowest reduction for all brands, failing to reach 60%.

### 3.2. Chemical Composition

All results show the means and standard deviations for control samples for each Brand, room temperature desalting, fridge temperature desalting, and heat desalting. Table 2 presents the chemical composition of all the samples studied (moisture, ashes, carbohydrates, proteins, and lipids).

The lowest mean moisture value was found for control Brand A (54.12 g/100 g), and the highest was for desalted samples at room temperature for Brand C (75.38 g/100 g). Control samples did not differ in moisture content across the three brands (*p* > 0.05). Room temperature and refrigerated desalting increased moisture levels, while heat desalting reduced the moisture content.

The highest ash content was observed in the control of Brand C (18.73 g/100 g), and the lowest was in the room temperature desalting of Brand B (3.87 g/100 g). All samples of all three brands (A, B and C) in all three desalting methods (room, refrigerated and heat), differed from each other, decreasing the ash content in accordance with the increase in the efficiency of the sodium removal, as shown in Table 2 of the chemical composition and Table 1 of the sodium content and percentage of sodium reduction.

**Figure 1:**
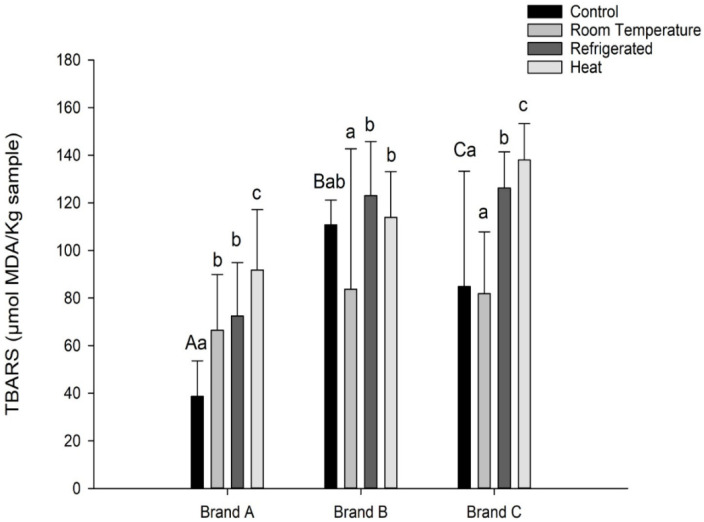
Mean and standard deviation of lipid oxidation expressed as TBARS µmol/kg for control and desalted samples for each Brand. Different uppercase letters for the control bars show a statistical difference. Different lowercase letters also show statistical difference within each brand to compare control and desalting techniques.

The highest average protein content was found in the heat desalting method of Brand B (33.49 g/100 g), and the lowest average was in the refrigerated desalting method of Brand C (16.38 g/100 g). The samples from all three brands (A, B, and C) at both room temperature and refrigerated desalting methods did not differ significantly from each other. They presented the lowest averages (*p* > 0.05). Figure 1 presents the lipid oxidation results for the control and desalted samples of the three brands.

The lowest average lipid values were found in the room temperature desalting of Brand C (1.37 g/100 g), and the highest average lipid values were found in the heat desalting of Brand A (4.19 g/100 g). The control samples from all three brands were significantly different in lipid content (*p* < 0.001), indicating heterogeneity in content.

The three brands presented different lipid oxidation levels in the control samples. The lowest lipid oxidation level was found in the control sample of brand A (38.63 µmol/kg), and the highest average lipid oxidation level was observed in brand C during the heat desalting process (138.02 µmol/kg), as shown in Figure 1. A significant increase in oxidation from room temperature to refrigerated desalting was observed in brands B (83.77 to 123.06) and C (81.88 to 126.28 µmol/kg), and a significant increase in brands A and C from refrigerated to heat desalting, respectively (A = 72.41 to 91.79 µmol/kg) and (C = 126.28 to 138.02 µmol/kg).

### 3.3. Determination of Titratable Acidity and pH

Table 3 presents titratable acidity and pH of jerked beef samples (control and desalted) from the three brands.

The room temperature desalting method for brand A had the lowest average titratable acidity (3.83), while the control sample for brand B had the highest average (11.79). There was a significant difference in acidity content between the three desalting methods for brands A and B. Only brand C showed no difference between the room temperature and refrigerated methods. The room-temperature desalting method had the lowest average titratable acidity, followed by the refrigerated method, while the heat method had the highest. This is confirmed by correlation with the lipid oxidation analyses, as shown in Figure 1. The samples with the highest titratable acidity also had the highest average lipid oxidation, while the samples with the lowest average titratable acidity had the lowest lipid oxidation index.

## 4. Discussion

### 4.1. Analysis of Sodium Content in Jerked Beef Samples

This study aimed to reproduce desalting techniques used in Brazilian restaurants to reduce the sodium content of jerked beef, a component of various everyday dishes on menus. The decision to have two water changes is based on the need to reduce water consumption during meal preparation and the feasibility of implementing this process within the restaurants. Additionally, the time for water change was determined by evaluating the sodium content in the desalting water in conjunction with the recommendation not to exceed 12 h at room temperature as described by AOAC [34]. Duarte [35] and Silva [36] recommend that desalting does not exceed 12 h, as extended periods at room temperature may favor microorganisms and compromise the safety and microbiological quality of the product. This study demonstrated very high sodium concentrations in the control samples (over 6000 mg/100 g), comparable to the values described by Sampaio et al. [37] for *charque* (≈5700 mg/100 g) and by Coró et al. [38] for jerked beef (≈5000–6000 mg/100 g). This confirms that both *charque* and jerked beef are meat products with critical sodium levels, reinforcing the need for desalting strategies to make them more suitable for human consumption from a public health perspective.

After the different desalting methods, it was observed that the room temperature technique was the most efficient, reducing sodium content by up to 76%, followed by refrigerated desalting (67–74%), and finally, heat desalting (52–58%). These results are consistent with [21], who reported reductions of nearly 90% in prolonged desalting, demonstrating that diffusion time and water exchange are determining factors in salt removal. The lower efficiency of heat desalting suggests that reduced contact time compromises diffusion, maintaining higher sodium levels. Comparing the studies, a pattern emerges: non-desalted products show sodium levels above 5000 mg/100 g. In contrast, products with controlled desalting can significantly reduce this value, approaching 2000 mg/100 g or lower with longer diffusion times.

Prolonged heat exposure of meat to improve sodium reduction would lead to further changes, including protein coagulation, lipid loss, and color changes. Therefore, temperature and time control likely reduced sodium loss into the water while preserving the structure of the jerked beef for subsequent cooking in restaurants. Desalting techniques not only reduce sodium but may also influence the chemical and physical composition of the samples.

Recent studies indicate that increased sodium intake is directly related to higher risks of hypertension, chronic kidney disease, and stroke [20,39]. Nilson et al. [20] estimate that an average reduction of 1 g of salt per day could prevent approximately 1.4 million cardiovascular deaths worldwide each year. Given this scenario, the application of technological interventions in traditional meat products, such as the controlled desalination of jerked beef, constitutes a relevant approach to reduce sodium content without compromising product quality. Such measures are aligned with global sodium reduction targets and contribute to maintaining the cultural and gastronomic identity of the food while promoting improved nutritional profiles and preventing cardiovascular diseases.

### 4.2. Chemical Composition

According to the data presented (Table 2), the moisture content of the control meat from the three brands complies with current Brazilian legislation, as IN 92/2020 establishes a maximum limit for this parameter of 60%, and the highest average obtained in this study was 58.5% [40]. The moisture content results showed significant variation across studies, reflecting both the type of product and the processing and desalting conditions. In the study by Sampaio et al. [37], Jerked beef had an average moisture content of 46%, a value slightly higher than *charque* (44%), suggesting greater water retention in this product. Coró et al. [38] confirmed the variability of this parameter, with values ranging from 39% to 60% according to the formulation and storage time, evidencing the influence of natural antioxidants and the reduction in curing salts on the composition. Correia and Biscontini [21] also observed higher amplitudes (46–69%), especially after desalting and cooking, indicating that the incorporation of water during these processes significantly increases the moisture content of the final product.

According to Fick’s First Law, which describes transport and mass transfer phenomena involving molecular diffusion due to concentration differences, diffusion occurs along a concentration gradient from the more concentrated to the less concentrated medium [41,42]. Therefore, given the results, it can be observed that this phenomenon occurred with statistical differences in desalting at room temperature and refrigerated temperatures. Similar data on increased moisture content during desalting at room temperature and refrigerated temperatures were reported by both Duarte [35] and Correia and Biscontini [21], which can be explained by the fact that, like this study, they performed desalting for 12 h. Regarding the reduction in moisture content due to heat desalting, the result can be explained by protein denaturation of the surface of the meat, which promotes water release from the tissue. This promotes fiber shrinkage and collagen solubilization, thereby reducing the moisture content [43]. Similar results were also obtained by Duarte [35]. The use of heat in the hot method expels more water from the meat, resulting in a drier texture. Thus, by integrating the studies, it is evident that the moisture content in jerked beef is highly dependent on the desalting method, ranging from less than 40% to more than 70%, with direct implications for texture, oxidative stability, and sensory acceptance.

For all batches of the three brands, the ash content of the control meat did not exceed 25%, within the legal limit [36]. Similar results were reported by Correia and Biscontini [21], who reported an average of 18.1%, and Duarte [35], who reported averages ranging from 16.2% to 18.4%. However, no standardization was observed in production, as evidenced by comparing the control meat from the three brands, which showed only statistical similarity between Brand A and Brand B. The ash values observed in the present study ranged from 16.98 to 18.73 g/100 g in the control samples, with significant reductions after desalting, especially at room temperature, resulting in values close to 4 g/100 g. This behavior confirms the efficiency of desalting in removing mineral salts, especially sodium, directly reflecting the ash content. Similar results were reported by Correia and Biscontini [21], who found marked ash reductions (up to 37%) after desalting and cooking, indicating that prolonged contact with water promotes significant salt diffusion. In the study by Coró et al. [38], ash values remained high (18–22%) due to the use of formulations with curing salts and antioxidant additives, without a desalting process, reinforcing the direct relationship between ash content and salt presence.

Compared with Sampaio et al. [37], who reported values of approximately 18% in *charque* and 16% in jerked beef, this study demonstrates that desalting can reduce ash content by up to 75%, especially when treated at room temperature. This reduction is consistent with the physicochemical principle of diffusion, in which a concentration gradient drives the expulsion of ions from the meat matrix into the aqueous medium. Thus, the different studies corroborate that the highest ash values are associated with non-desalted products or those formulated with additional salts. In contrast, the lower values observed in this study reflect the desalting efficiency and the consequent reduction in sodium and other minerals in meat.

Heat-desalted samples showed the highest protein averages across all three brands, exceeding the averages for the control samples. This is explained by variations in the solid components of the samples relative to their total composition. The sodium content removed from the samples by the desalting methods offsets the results for other solids, such as protein and lipid contents, thereby raising their values in the heat-desalted samples, with moisture percentages comparable to those of the control. For the samples from the ambient and refrigerated desalting methods, the values found are lower than those of the control for all three brands. Although they lost sodium, as with the heat-desalted method, the moisture content was high, ranging from 73 to 75 g/100 g, thus diluting the samples’ protein content.

The protein results obtained in this study ranged from 22 to 24 g/100 g in the control samples, which are lower than those observed by Coró et al. [38], who reported values ranging from 27 to 33%, and those reported by Sampaio et al. [37], who found approximately 28% for jerked beef. Correia and Biscontini [21] observed wider ranges, between 21 and 35%, depending on the processing and the desalting or cooking stage. This variation may be associated with both the initial composition of the raw material and the effect of technological processes, especially moisture and salt management.

In this study, desalting at room temperature or under refrigeration resulted in a significant reduction in protein content (16–19%), attributed to dilution caused by increased moisture. In contrast, heat desalting resulted in higher values (31–33%), close to the upper limit reported by Correia and Biscontini [21], reflecting the relative concentration of solids due to lower water absorption. These results reinforce the importance of the methodology used to determine protein and demonstrate that protein levels in jerked beef are not static, but vary according to the desalting method, corroborating previous observations that moisture and salt manipulation directly impact proximate composition.

Similar results were reported in a study by Duarte [35], which found an initial protein content of 24.95 g/100 g. After 12 h of desalting, reductions of 23.9% were recorded at room temperature and 7.5% under refrigeration. After 24 h, the reductions reached 31.5% and 5.0%, respectively. These findings were attributed to the “salting in” effect, which increases protein solubility and promotes its dispersion in the desalting water. When the process was combined with cooking, a relative increase in protein was observed, which can be explained by the concentration of solids after moisture loss. Thus, convergence is observed between the present work and the literature, confirming that desalting conditions and subsequent thermal processing strongly modulate the dynamics of protein content in jerked beef.

The control samples of all three brands differed significantly in lipid content, demonstrating heterogeneity. This is explained by the differences in the interspersed layers of meat and fat in the different brands. An increase in the average lipid content, particularly in the heat-desalted process, may have occurred for the same reason mentioned above for protein. With the removal of sodium and the reduction in ash and moisture content, the lipid content increases proportionally. The lipid content varied considerably depending on the desalting method and Brand. The lowest average was observed in the room temperature desalting of brand C (1.37 g/100 g), while the highest value was found in the heat desalting of brand A (4.19 g/100 g). These results partially contrast with those reported by Sampaio et al. [37], who observed lipid values of around 10% for jerked beef and around 21% for *charque*. The difference can be attributed to the type of cut, initial fat content, and the absence of desalting in the study by Sampaio et al. [37], which results in higher total solids, including lipids. Coró et al. [38] also reported a wide variation (2 to 9%), depending on the formulation with nitrite or natural antioxidants, a range closer to that found in the present study, suggesting that technological factors (addition of salts—sodium and nitrate—and extracts—oregano, rosemary—in addition to moisture manipulation—drying, cure and desalting) directly affect the lipid proportion. These technological variables alter osmotic pressure, solute diffusion, and lipid oxidative stability, thereby significantly affecting the physicochemical composition and structural behavior of dried meat and similar products.

Correia and Biscontini [21] reported lipid contents ranging from 3 to 14%, depending on the desalting and cooking stages. These results aligned with those observed in the current study, where desalting resulted in apparent reductions in lipid content in methods with higher water absorption (room and refrigerated), but led to a relative increase in lipid content in heating methods due to the concentration of solids. This dilution/concentration effect reinforces the central role of moisture and ash in the proximate composition; when these components decrease, lipids become relatively more significant in the total percentage. Taken together, the four studies demonstrate that lipid levels in jerked beef can vary from 1.3% to over 10%, depending on the formulation, desalting stage, and methodology. In this study, the heterogeneity among commercial brands highlights the importance of the initial raw material. At the same time, comparison with the literature shows that higher values (such as the 10% reported by Sampaio et al. [37]) are associated with non-desalted products, while lower values reflect both salt reduction and moisture increase processes, as well as differences in the cut. Therefore, lipid variation should be interpreted in light of the interaction between meat composition, the desalting process, and analytical methodology, which collectively explain the breadth of the observed results.

Similar results were reported in the study by Duarte [35], who found an initial lipid content of 6.74 g/100 g. After 12 h of desalting, reductions of 9.9% were observed at room temperature and 20.6% under refrigeration. After 24 h, the reductions reached 27.1% and 23.1%, respectively. These findings were attributed to dilution caused by the incorporation of water during desalting, a mechanism also observed in the present study for both the room temperature and refrigerated methods.

The lowest average carbohydrate values found were in the refrigerated desalting method of brand A (0.10 g/100 g), and the highest average was in the heat desalting method of brand A (2.69 g/100 g). With the effect of the variation in sodium, protein, and lipid contents between the three desalting methods, the carbohydrate contents also varied due to the proportionality of the solids in the total composition of the meat, since the carbohydrate content is calculated based on the difference in content between the other solids in the product. The significant reduction in total carbohydrates in the refrigerated desalting of Brand A may have occurred due to the increase in moisture content, compared with the brand control (control moisture: 54.12 g/100 g; refrigerated moisture: 73.34 g/100 g).

When comparing the studies [21,35,37,38], convergence is observed. All reported residual carbohydrates were at very low levels (<2%). In Coró et al. [38], a variation of 0.5 to 1.5% was associated with different antioxidant formulations, while Correia and Biscontini [21] observed similar values in *charque* and jerked beef under different desalting and cooking treatments. This pattern was also confirmed [35], noting that jerked beef contains virtually no relevant carbohydrates and that the reported values are due to calculation by difference. Thus, both the current and previous studies confirm that carbohydrates do not represent a significant fraction of the composition of jerked beef and that the variability found is mainly due to the analytical methodology and changes in moisture and salts during processing.

When comparing the studies, a point of convergence is observed: moisture content and the presence of additives (such as nitrite) play a determining role in the susceptibility of jerked beef to lipid oxidation. In the study by Sampaio et al. [37], jerked beef showed higher moisture and nitrite content than *charque*, leading to greater oxidative instability.

There was a significant difference in the mean lipid oxidation values between the control meat and the three brands. This significant variation may be directly related to the different chemical compositions of the beef used in the curing process, including the nitrate and nitrite levels, as well as the different unit operations used in processing this meat across the various brands, such as the use of the time/temperature thermal process binomial, among others.

Mediani et al. [44] reported that lipid oxidation causes significant changes during food storage and manufacturing, leading to rancidity. Another important factor to consider regarding the differences in lipid oxidation levels found in control meats from different brands is the varying storage conditions of the product after processing in the industry. It is known that the oxygen atmosphere, the storage temperature of the processed product, and the product’s water content are key factors that affect lipid oxidation reactions, compromising the initial quality of the product before any desalting method is employed.

The tendency for increased lipid oxidation in the heat desalting method compared to the other two methods was expected, given that heat is a primary factor in accelerating the lipid oxidation reaction, which occurs at temperatures similar to those used in desalting. Thermal degradation occurs through direct heat action, promoting protein denaturation, coagulation, water loss, and partial cleavage of lipid bonds, phenomena intensified at temperatures above 60 °C and dependent on exposure time [45]. Oxidative degradation, on the other hand, results from the reaction of free radicals with unsaturated fatty acids, generating peroxides, aldehydes, and ketones that compromise the aroma, color, and nutritional value of the meat [46]. During heat desalting, these mechanisms can occur simultaneously, as the increase in temperature accelerates the diffusion of sodium and the release of lipids, but also intensifies lipid oxidation, reducing the oxidative stability and sensory quality of the final product. In this context, controlling technological parameters, especially temperature, heating time, and humidity, is essential to minimize chemical changes and preserve the structural integrity of jerked beef.

A curious finding was the increase in lipid oxidation during refrigerated desalting, as refrigeration slows oxidation by reducing reaction rates. Wazir et al. and Xia, Zhou, and Xu [47,48] found that high temperatures accelerate lipid oxidation reactions in meat products, promoting the formation of primary and secondary oxidation products, including hydroperoxides and aldehydes, as they are precursors of compounds that cause rancidity and oxidation. Studies by Shimizu and Shimizu and by Rahman, Al-Khusaibi, and Guizani [49,50] indicate that lower temperatures delay oxidation, thereby reducing the intensity of chemical reactions. However, the results suggest that factors such as meat composition, the presence of pro-oxidants, and the duration of meat exposure to water play critical roles in the kinetics of lipid oxidation during heat and cold desalting processes. Thus, the higher acidity and oxidation observed in the control sample of brand B underscore the importance of adequately controlling the raw material and processing steps to ensure the stability and quality of meat products. On the other hand, curing salts such as nitrite are known to prevent lipid oxidation in meat products, acting as antioxidants that inhibit free radical chain reactions and delay rancidity, a reaction considered one of the main functions of nitrite salts in meat processing. Therefore, the amount of nitrite may have varied considerably in the samples according to each desalting method, thus varying lipid oxidation between the desalting methods [51].

Comparing these findings with those of Sampaio et al. [37], it is observed that the oxidation found in jerked beef (≈1.0 mEq/kg) is consistently higher than in *charque* (≈0.35 mEq/kg). This confirms the greater susceptibility of jerked beef, associated with its higher moisture content and lower salt concentration. The results of the present study are consistent with this interpretation, since desalting at room temperature, by promoting greater water input and ash reduction, increases the oxidative tendency, although to a lesser extent than the heat method.

Data from Coró et al. [38] further reinforces this scenario, showing that, under storage conditions, jerked beef without antioxidants can reach TBARS levels above 4 mg MDA/kg after 60 days. However, the addition of natural antioxidants, such as yerba mate and propolis, was effective in reducing these values to ranges between 1.6 and 2.1 mg MDA/kg, similar to those found in nitrite-treated products. This comparison suggests that although the desalting process affects oxidation in the short term, additional strategies, such as antioxidant use, may be necessary to maintain stability throughout the shelf life.

The study by Correia and Biscontini [21] showed that desalting and cooking processes also intensify lipid oxidation, although without numerical details. This finding aligns with the present study, which found that heat desalting produced the highest levels of TBARS, confirming that heat accelerates the formation of secondary oxidation products. Thus, the literature supports the observation that both salt diffusion and heat application are key factors in determining the oxidative stability of jerked beef.

### 4.3. Determination of Titratable Acidity and pH

The control sample from brand B presented the highest level of titratable acidity. It showed the highest level of initial lipid oxidation, even before any desalting method was used, suggesting nonconformities in the production and/or quality control of that Brand. This is explained by the formation of chemicals such as peroxides, resulting from lipid oxidation, as well as the release of free fatty acids in the meat, also a result of lipid oxidation, which leads to increased titratable acidity.

Dominguez et al. and Amaral, Silva, and Lannes [46,52] emphasize that lipid oxidation is a process involving multiple mechanisms with complex reactions and interactions between substrates and catalysts. These reactions are influenced by various factors, leading to the formation of peroxides and byproducts that affect the food’s chemical stability, increase acidity, and influence the sensory and nutritional quality of the meat. However, Mediani et al. and Rahman et al. [44,50] state that the drying and storage process intensifies lipid oxidation, resulting in the accumulation of peroxides and aldehydes that directly impact meat stability and are a determining factor in the development of rancidity, consequently leading to a loss of technological and sensory quality in meat and meat products. Furthermore, studies by Wazir et al. [47] reinforced the notion that ready-to-eat meat products with higher peroxide values also showed increased free fatty acid content, indicating a direct relationship between lipid oxidation and acidity.

Compared to literature, the results are consistent with those reported by [34], who found average values of around 0.7 g/100 g, with jerked beef presenting higher acidity than *charque* [38]. Moreover, they reported similar ranges (0.5–0.8 g/100 g), regardless of the partial replacement of nitrite with natural antioxidants, suggesting that acidity is relatively stable even with formulation changes. Ref. [19] identified ranges of 0.4–0.7 g/100 g, close to those observed in the present study, especially after desalting.

Similar results were reported by Correia and Biscontini [21], who, when evaluating different methods of desalting jerked beef, highlighted that the variation in titratable acidity remained within narrow ranges, from 0.4 to 0.8 g/100 g, reflecting the effect of dilution and moisture balance rather than intense biochemical transformations. The author also emphasized that, although the pH did not present significant variations, its stability is fundamental for the safety of the product, since adequate acidity and pH values help limit microbial growth during desalting.

Despite the relevant results, this study has limitations, as it was conducted under controlled laboratory conditions and evaluated only one type of cut. Therefore, future studies should investigate the kinetics of sodium diffusion, as well as the microstructural and microbiological changes and the sensory impact of desalting under different times and temperatures, in addition to exploring the use of complementary technologies, such as ultrasound and natural antioxidants, to optimize sodium removal and consequently preserve the quality of cured meat.

## 5. Conclusions

Jerked beef contains high amounts of sodium, exceeding 6000 mg/100 g, making it a product that requires desalting before being added to various dishes. Desalting in room-temperature water is the most efficient method for reducing sodium in jerked beef, even though final levels remain high compared to recommended daily intakes. This reinforces the need to standardize the production process and the policies for reformulating meat products.

It is noteworthy that, although the sodium content remains above the WHO-recommended intake limits, the desalted product shows a significant reduction compared to the control and can be used as an intermediate ingredient in culinary preparations (such as “feijoada”, casseroles, fillings, and typical regional dishes). In these applications, additional dilution of the sodium content during cooking contributes to the product’s suitability for a diet with lower sodium. This approach enhances the technological and gastronomic viability of desalted, dried meat without compromising the product’s cultural identity.

In this study, desalting at room temperature proved to be more effective, reducing sodium with a lower increase in TBARS. While the heat method substantially increases oxidative instability, acidity is less impacted by desalting conditions than other components (such as sodium, protein, and lipids). However, it may still reflect compositional changes resulting from salt and water balance. Therefore, this study demonstrated that the desalting method significantly influences not only sodium reduction but also the nutritional composition, acidity, pH, and oxidative stability of the jerked beef product.

## Figures and Tables

**Table 1:** Sodium content of various brands of salted and desalted Jerked beef, along with the percentage reduction of sodium compared to control (salted) samples.

	Control	Room Temperature		Refrigerated		Heat	
		Mean ± SD (mg de Na/100 g)	% Reduction	Mean ± SD (mg de Na/100 g)	% Reduction	Mean ± SD (mg de Na/100 g)	% Reduction
Brand A	6339.2 ± 317.2 ^cA^	1527.4 ± 154.2 ^a^	75.9	1643.0 ± 293.5 ^a^	74.1	2676.3 ± 400.9 ^b^	57.8
Brand B	6538.6 ± 415.6 ^dA^	1565.8 ± 190.3 ^a^	76.1	1946.8 ± 273.6 ^b^	70.2	3148.7 ± 454.2 ^c^	51.8
Brand C	6459.8 ± 465.7 ^dA^	1653.6 ± 162.9 ^a^	74.4	2122.4 ± 267.8 ^b^	67.1	2922.8 ± 385.1 ^c^	54.8

The same lowercase letters in the same row represent no statistical difference. The same uppercase letters in the same column represent no statistical difference. Uppercase letters were only included to compare control samples from different Brands.

**Table 2:** Food analyses (g/100 g) of control and desalted Jerked beef.

	Control	Room Temp.	Refrigerated	Heat
		Brand A		
Moisture	54.12 ± 1.24 ^Aa^	73.82 ± 1.57 ^b^	73.34 ± 1.70 ^b^	54.48 ± 2.23 ^a^
Ashes	16.98 ± 0.73 ^Ad^	3.95 ± 0.49 ^a^	4.36 ± 0.81 ^b^	7.22 ± 0.88 ^c^
Carbohydrate	1.64 ± 0.84 ^Ab^	0.56 ± 0.54 ^ab^	0.10 ± 0.06 ^a^	2.69 ± 0.69 ^c^
Protein	23.50 ± 1.39 ^Ab^	18.70 ± 2.11 ^a^	18.07 ± 1.40 ^a^	31.29 ± 2.82 ^c^
Lipids	3.70 ± 0.98 ^Aa^	3.00 ± 0.62 ^b^	3.94 ± 0.90 ^ac^	4.19 ± 1.51 ^c^
		Brand B		
Moisture	56.44 ± 2.57 ^Aa^	75.21 ± 2.05 ^c^	73.80 ± 2.38 ^d^	54.42 ± 2.52 ^b^
Ashes	17.15 ± 0.68 ^Ad^	3.87 ± 0.28 ^a^	4.92 ± 0.47 ^b^	8.76 ± 0.78 ^c^
Carbohydrate	0.47 ± 0.34 ^Aa^	0.55 ± 0.37 ^a^	2.14 ± 0.97 ^b^	0.35 ± 0.09 ^a^
Protein	23.99 ± 3.22 ^Ab^	18.08 ± 1.95 ^a^	17.69 ± 2.86 ^a^	33.49 ± 3.09 ^c^
Lipids	1.97 ± 0.73 ^Bb^	2.40 ± 0.71 ^c^	1.62 ± 0.53 ^a^	3.46 ± 0.77 ^d^
		Brand C		
Moisture	56.07 ± 0.67 ^Ab^	75.38 ± 2.37 ^c^	74.83 ± 1.84 ^c^	54.26 ± 1.23 ^a^
Ashes	18.73 ± 0.43 ^Bd^	4.74 ± 0.59 ^a^	6.00 ± 0.53 ^b^	8.05 ± 0.77 ^c^
Carbohydrate	0.31 ± 0.48 ^Aa^	0.88 ± 0.80 ^a^	1.30 ± 0.30 ^a^	1.62 ± 0.78 ^a^
Protein	22.23 ± 1.27 ^Bb^	16.92 ± 1.14 ^a^	16.38 ± 1.13 ^a^	33.34 ± 1.26 ^c^
Lipids	2.85 ± 1.32 ^Cb^	1.37 ± 0.94 ^a^	1.48 ± 0.68 ^a^	2.71 ± 0.57 ^b^

The same lowercase letters in the same row represent no statistical difference. Different uppercase letters in the same column for each chemical analysis show a statistical difference. Uppercase letters were only included to compare control samples from different Brands.

**Table 3:** Mean and standard deviation for pH and acidity levels for brands A, B, and C of jerked beef (control and after desalting).

	Control	Room Temperature	Refrigerated	Heat
Brand A				
Acidity (g/100 g)	8.82 ± 1.29 ^d^	3.83 ± 1.36 ^a^	4.78 ± 1.20 ^b^	5.54 ± 1.37 ^c^
pH	5.83 ± 0.20 ^a^	6.01 ± 0.27 ^b^	6.03 ± 0.39 ^b^	5.82 ± 0.23 ^a^
Brand B				
Acidity (g/100 g)	11.79 ± 1.85 ^d^	6.47 ± 1.48 ^a^	6.94 ± 1.09 ^b^	9.75 ± 0.87 ^c^
pH	5.61 ± 0.20 ^a^	5.64 ± 0.17 ^a^	5.63 ± 0.19 ^a^	5.76 ± 0.10 ^b^
Brand C				
Acidity (g/100 g)	10.94 ± 0.99 ^c^	5.67 ± 0.69 ^a^	5.53 ± 0.49 ^a^	8.28 ± 2.57 ^b^
pH	5.89 ± 0.11 ^a^	5.76 ± 0.05 ^b^	5.82 ± 0.04 ^c^	5.86 ± 0.05 ^a^

The same letters in the same row represent no statistical difference.

## Data Availability

The data presented in this study are available on request from the corresponding author.
